# Cost-Effectiveness Analysis of Nivolumab Plus Ipilimumab for Advanced Non-Small-Cell Lung Cancer

**DOI:** 10.3389/fphar.2021.580459

**Published:** 2021-08-23

**Authors:** Xiaomin Wan, Xiaohui Zeng, Liubao Peng, Ye Peng, Qiao Liu, Lidan Yi, Xia Luo, Qijian Deng, Chongqing Tan

**Affiliations:** ^1^ Department of Pharmacy, The Second Xiangya Hospital of Central South University, Changsha, China; ^2^ PET-CT Center, The Second Xiangya Hospital of Central South University, Changsha, China; ^3^ Department of Psychiatry, The Second Xiangya Hospital, Central South University, Changsha, China; ^4^ Hunan Key Laboratory of Psychiatry and Mental Health, Chinese National Clinical Research Center on Mental Disorders (Xiangya), Mental Health Institute of the Second Xiangya Hospital, Chinese National Technology Institute on Mental Disorders, Central South University, Changsha, China

**Keywords:** nivolumab plus ipilimumab, chemotherapy, cost-effectiveness, non-small cell lung cancer, Markov model

## Abstract

**Objective:** This study evaluated the cost-effectiveness of nivolumab plus ipilimumab vs. chemotherapy in the first-line setting for patients with advanced non-small-cell lung cancer (NSCLC) from the US payer perspective.

**Materials and methods:** A Markov model wasdeveloped to evaluate the cost and effectiveness of nivolumab plus ipilimumab vs. chemotherapy in the first-line treatment of advanced NSCLC. The survival benefits of nivolumab plus ipilimumab were based on the results of the CheckMate 227 trial. The main endpoints of the model were cost, life-years (LYs), quality-adjusted LYs (QALYs), and incremental cost-effectiveness ratio (ICER). Univariable and probabilistic sensitivity analyses were conducted to assess model uncertainty. Additonal subgroup analyses were also performed.

**Results:** nivolumab plus ipilimumab produced a gain of 0.62 QALYs, at a cost of $104238 per QALY. The variables that had the greatest influence on the ICER were body weight and overall survival (OS) hazard ratio (HR). The probability of nivolumab plus ipilimumab being cost-effectiveness compared to chemotherapy is 50.7 and 66.2% when the willingness-to-pay (WTP) value is $ 100,000 and $ 150,000 per QALY. The results of subgroup analyses showed the ICER remained below $150,000/QALY regardless of the PD-L1 expression level.

**Conclusions:** nivolumab plus ipilimumab was estimated to be cost-effective compared with chemotherapy for patients with advanced NSCLC at a WTP threshold from 100,000/QALY to 150,000/QALY.

## Introduction

Lung cancer is the leading cause of cancer-related deaths worldwide, accounting for 18.4% of all cancers in 2018 (Bray et al., 2018). In the United States, there were an estimated 222,500 newly diagnosed lung cancer cases in 2017 (Siegel et al., 2017), of which 80–85% were non-small-cell lung cancer (NSCLC) cases (American Cancer Society (2020).

The standard first-line treatment for advanced NSCLC without known targetable drive mutation is platinum doublet chemotherapy, but there is nooverall survival (OS) benefit (Reck et al., 2019). Recently, the introduction of immune checkpoint inhibitors has greatly improved the prognosis of NSCLC (Topalian et al., 2015). Currently, two types of checkpoint inhibitors have been approved for cancer treatment. One is to inhibit the CD28/CTLA-4 system of immune modulation, such as ipilimumab, and the other is to inhibit the interaction between programmed death 1 (PD-1) and programmed cell death 1 ligand 1(PD-L1), such as atezolizumab, avelumab, durvalumab, nivolumab, and pembrolizumab (Reck et al., 2019).

CTLA-4 functions during the priming phase of T-cell activation, while PD-L1 functions during the effector phase of the tumor microenvironment. To provide effective first-line treatment for a wider patient population, the CheckMate 227 trial (Hellmann et al., 2019) evaluated the efficacy of the PD-1 inhibitor nivolumab combined with the anti-CTLA-4 antibody ipilimumab as the first-line treatment for advanced NSCLC. The results showed that the OS of nivolumab plus ipilimumab was longer than chemotherapy regardless of the PD-L1 expression level (hazard ratio [HR], 0.73; 95% CI, 0.64–0.84). Nivolumab plus ipilimumab also reduced grade 3 and 4 treatment-related adverse events compared to chemotherapy (32.68 vs. 36.0%).

The purpose of this study was to evaluate the cost-effectiveness of nivolumab plus ipilimumab versus chemotherapy as the first-line treatment for patients with advanced NSCLC from the US payer perspective.

## Materials and Methods

**FIGURE 1:**
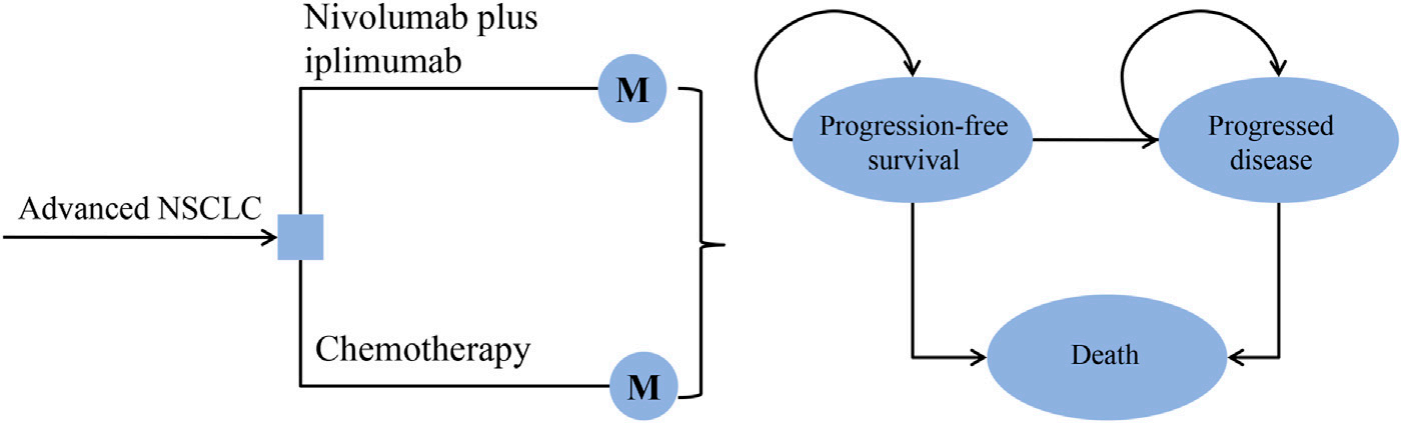
The decision tree and the Markov state transition model. NSCLC: non-small-cell lung cancer.

A Markov model was developed to evaluate the cost and effectiveness of nivolumab plus ipilimumab vs. chemotherapy as the first-line treatment for patients with advanced NSCLC (Figure 1). This economic evaluation used a mathematical model to simulate patients, so there was exempt from Institutional Review Board approval. It was assumed that all patients received first-line treatment until disease progression, and both groups could receive second-line treatment until death.

The time horizon of the model simulation waslifetime. Each cycle represented 6 weeks. A half-cycle correction was applied. The main endpoints of the model were cost, life-years (LYs), quality-adjusted LYs (QALYs), and incremental cost-effectiveness ratio (ICER). Only direct medical costs were considered. Both costs and outcomes were adjusted at a discount rate of 3% per year. The Markov model was implemented in TreeAge Pro 2011 software (https://www.treeage.com/), and statistical analyses were performed in R software (http://www.r-project.org).

### Model Survival and Progression Risk Estimates

The survival benefits of nivolumab plus ipilimumabwere based on the results of the CheckMate 227 trial. The overall probability of death included the probability of death from advanced NSCLC and background mortality rate from other causes. The probability of death and risk of progression were derived from the OS and progression-free survival (PFS) curves published in the CheckMate 227 trial (Hellmann et al., 2019). Data points were extracted from published survival curves by using GetData Graph Digitizer software (http://www.getdata-graph-digitizer.com/index.php), and then Pseudo-individual patient data was generated according to the method of Hoyle and Henley (2011). According to the Akaike information criterion, we found that the log-logistic model has a good fit for all curves. The background mortality rate was obtained from US life tables (Arias et al., 2017) (Supplemental Table 1).

### Cost Estimates

We only considered direct costs and adjust costs to 2020 US dollars using the US Consumer Price Index (Department of Labor, 2020). Direct medical costs included drug, administration, and management of adverse effects (AEs) costs. The unit price of drugs was estimated based on the average wholesale price of the Centers for Medicare and Medicaid Services in 2020 (rug Pricing Files, 2020) (Table 1). AE costs were derived from previously published studies (Ting et al., 2015; Wan et al., 2019a). The administration costs were calculated based on the Medicare physician fee schedule in 2020 (Table 1) (Centers for Medicare and Me, 2020).

**TABLE 1:** Model parameters.

Variable	Baseline value	Range		Distribution
		Minimum	Maximum	
HR of NIVO vs. chemotherapy for PFS	0.79 (Hellmann et al., 2019)	0.69	0.91	Normal
Log-logistic PFS survival model with chemotherapy	λ = 0.1937502,γ = 1.950076	—	—	—
HR of NIVO vs. chemotherapy for OS	0.73 (Hellmann et al., 2019)	0.64	0.84	Normal
Log-logistic OS survival model with chemotherapy	λ = 0.07476837,γ = 1.52037	—	—	—
NIVO AEs incidence				
Diarrhea	0.170 (Hellmann et al., 2019)	0.136	0.204	Beta
Rash	0.170 (Hellmann et al., 2019)	0.136	0.204	Beta
Fatigue	0.144 (Hellmann et al., 2019)	0.115	0.173	Beta
Nausea	0.099 (Hellmann et al., 2019)	0.079	0.119	Beta
Anemia	0.038 (Hellmann et al., 2019)	0.030	0.046	Beta
Neutropenia	0.002 (Hellmann et al., 2019)	0.0016	0.0024	Beta
Chemotherapy AEs incidence				
Diarrhea	0.096 (Hellmann et al., 2019)	0.077	0.115	Beta
Rash	0.053 (Hellmann et al., 2019)	0.042	0.064	Beta
Fatigue	0.189 (Hellmann et al., 2019)	0.151	0.227	Beta
Nausea	0.361 (Hellmann et al., 2019)	0.289	0.433	Beta
Anemia	0.330 (Hellmann et al., 2019)	0.264	0.396	Beta
Neutropenia	0.172 (Hellmann et al., 2019)	0.138	0.206	Beta
Utility (SD)				
Progression-free disease	0.71 (0.24) (Chouaid et al., 2013)	0.57	0.85	Beta
Progressed disease	0.67 (0.20) (Chouaid et al., 2013)	0.54	0.80	Beta
AEs disutility				
Diarrhea	−0.320 (Nafees et al., 2017)	−0.256	−0.384	Beta
Rash	−0.150 (Nafees et al., 2017)	−0.120	−0.180	Beta
Fatigue	−0.410 (Nafees et al., 2017)	−0.328	−0.492	Beta
Nausea	−0.250 (Nafees et al., 2017)	−0.200	−0.300	Beta
Anemia	−0.073 (Wan et al., 2019a)	−0.058	−0.088	Beta
Neutropenia	−0.460 (Nafees et al., 2017)	−0.368	−0.552	Beta
Patients’ weight, kg	70 (Wan et al., 2019a)	60	140	Norm
Drug cost, US$				
nivolumab/mg	29.345 (rug Pricing Files (2, 2020)	23.476	35.214	Lognormal
ipilimumab/mg	161.70 (rug Pricing Files (2, 2020)	129.36	194.04	Lognormal
Gemcitabine/200 mg	4.331 (rug Pricing Files (2, 2020)	3.4648	5.1972	Lognormal
Pemetrexed/10 mg	73.766 (rug Pricing Files (2, 2020)	59.0128	88.5192	Lognormal
Cisplatin/10 mg	2.010 (rug Pricing Files (2, 2020)	1.608	2.412	Lognormal
Subsequent therapy cost in NIVO arm	1858 (rug Pricing Files (2, 2020)	1,486	2,230	Lognormal
Subsequent therapy cost in Chemotherapy arm	8,448 (rug Pricing Files (2, 2020)	6,758	10,138	Lognormal
AEs cost, US$				
Diarrhea	16,510 (Wan et al., 2019a)	13,208	19,812	Lognormal
Rash	7,872 (Wan et al., 2019a)	6,298	9,446	Lognormal
Fatigue	0 (Wan et al., 2019a)	0	0	-
Nausea	2,586 (Ting et al., 2015)	2069	3,103	Lognormal
Anemia	20,260 (Wan et al., 2019a)	16,208	24,312	Lognormal
Neutropenia	17,181 (Wan et al., 2019a)	13,745	20,617	Lognormal
Administration cost, US$				
First hr	143.08 (Centers for Medicare & Me, 2020)	114.46	171.70	Lognormal
Additional hr	30.99 (Centers for Medicare & Me, 2020)	24.79	37.19	Lognormal

HR, hazard ratio; NIVO, nivolumab + ipilimumab; PFS, progression-free survival; OS, overall survival; AEs, adverse effects.

In first-line treatment, the drug costs of chemotherapy were based on the following regimen (Hellmann et al., 2019): chemotherapy for patients with nonsquamous NSCLC included pemetrexed 500 mg/m^2^ plus cisplatin 75 mg/m^2^, once every 3 weeks for up to four cycles; For patients with squamous NSCLC, chemotherapy included gemcitabine 1250 mg/m^2^ plus cisplatin 75 mg/m^2^, once every 3 weeks, with a maximum of four doses; After four doses, patients with nonsquamous NSCLC could be maintained with pemetrexed (500 mg/m^2^) until the disease progresses. The drug costs of immunotherapy were based on the following regimen: nivolumab and ipilimumab were administered with 3 mg/kg every 2 weeks and 1 mg/kg every 6 weeks, respectively. According to the observations of the CheckMate 227 trial, 44% of patients in the nivolumab plus ipilimumab arm and 56% of patients in the chemotherapy arm received subsequent systemic therapy; docetaxel, nivolumab, pembrolizumab, and ipilimumab were the most used therapies (Hellmann et al., 2019). The body surface area of 1.86 m^2^ and a bodyweight of 70 kg were used to calculate the drug dose (Goulart and Ramsey, 2011). The model considered the effects of grade 1 or grade 2 and grade 3 or grade 4 AEs, including fatigue, diarrhea, rash, nausea, anemia, and neutropenia (Hellmann et al., 2019) (Table 1).

### Utility Estimates

Each health state was assigned a health utility value (Table 1). The utility of perfect health is valued 1 and dead is valued 0. Since the health-related quality of life was not reported in the CheckMate 227 trial, baseline utility estimates for PFS and progressed disease (PD) health states and utility values for AEs were obtained from previously published studies based on patients with NSCLC. The utilities of the patients with PFS and PD state we used were 0.71 and 0.67, respectively, (Chouaid et al., 2013). Due to a lack of quality of life data, we did not consider the different utility of each treatment arm. However, we considered the disutility of AEs according to the methods of Anna Oh et al (2017).

### Sensitivity Analysis

A series of sensitivity analyses were performed to assess the robustness of the model and the uncertainty in parameter estimation. In the univariable sensitivity analysis, we varied the value of one parameter at a time and make it varied within ±20% of the baseline value to explore the impact of each parameter on ICER. In the probabilistic sensitivity analyses, 1,000 Monte Carlo simulations were performed on a random sample of the distribution of all parameters simultaneously.

We also considered the subgroup of patients for patients with a PD-L1 expression level of <1%, ≥1%, or ≥50% in the CheckMate 227 trial. For these subgroups, we assumed the same data as for all subgroups in the trial except for the HR where there was not enough data. The subgroup-specific HRs were listed in Table 2.

**TABLE 2:** Results for subgroup analyses.

Subgroup	Sample size		OS HR (95% CI)	PFS HR (95%CI)	ICER	Cost-effectiveness probability at the threshold	
	Nivolumab + ipilimumab	Chemotherapy				$100,000/QALY	$150,000/QALY
PD-L1 ≥1%	396	397	0.79 (97.72% CI, 0.65–0.96)	0.82 (95% CI, 0.69–0.97)	128948	43.5%	57.3%
PD-L1 ≥50%	205	192	0.70 (95% CI, 0.55–0.90)	0.62 (95% CI, 0.49–0.79)	126910	39.1%	59.7%
PD-L1 <1%	187	186	0.62 (95% CI, 0.48–0.78)	0.75 (95% CI, 0.59–0.96)	77,040	66%	87.3%

OS, overall survival; HR, hazard ratio; PFS, progression-free survival; ICER, incremental cost-effectiveness ratio; QALY, quality-adjusted life-years.

## Results

### Base Case Results

The baseline analysis results of the model are listed in Table 3. The use of nivolumab plus ipilimumab compared with chemotherapy produced a gain of 1.11 LYs. Accounting for quality of life, patients receiving nivolumab plus ipilimumab produced a gain of 0.62 QALYs. The ICER for nivolumab plus ipilimumab compared with chemotherapy was $104,238 per QALY.

**TABLE 3:** base case results.

Results	Nivolumab plus iplimumab	Chemotherapy	Incremental
Life-years	3.12	2.01	1.11
QALYs	1.88	1.26	0.62
Total cost, $	236795	171577	65,218
ICER	—	—	—
Per life-year	—	—	58,661
Per QALY	—	—	104238

ICER: incremental cost-effectiveness ratio; QALY: quality-adjusted life-years.

### Sensitivity Analysis

**FIGURE 2:**
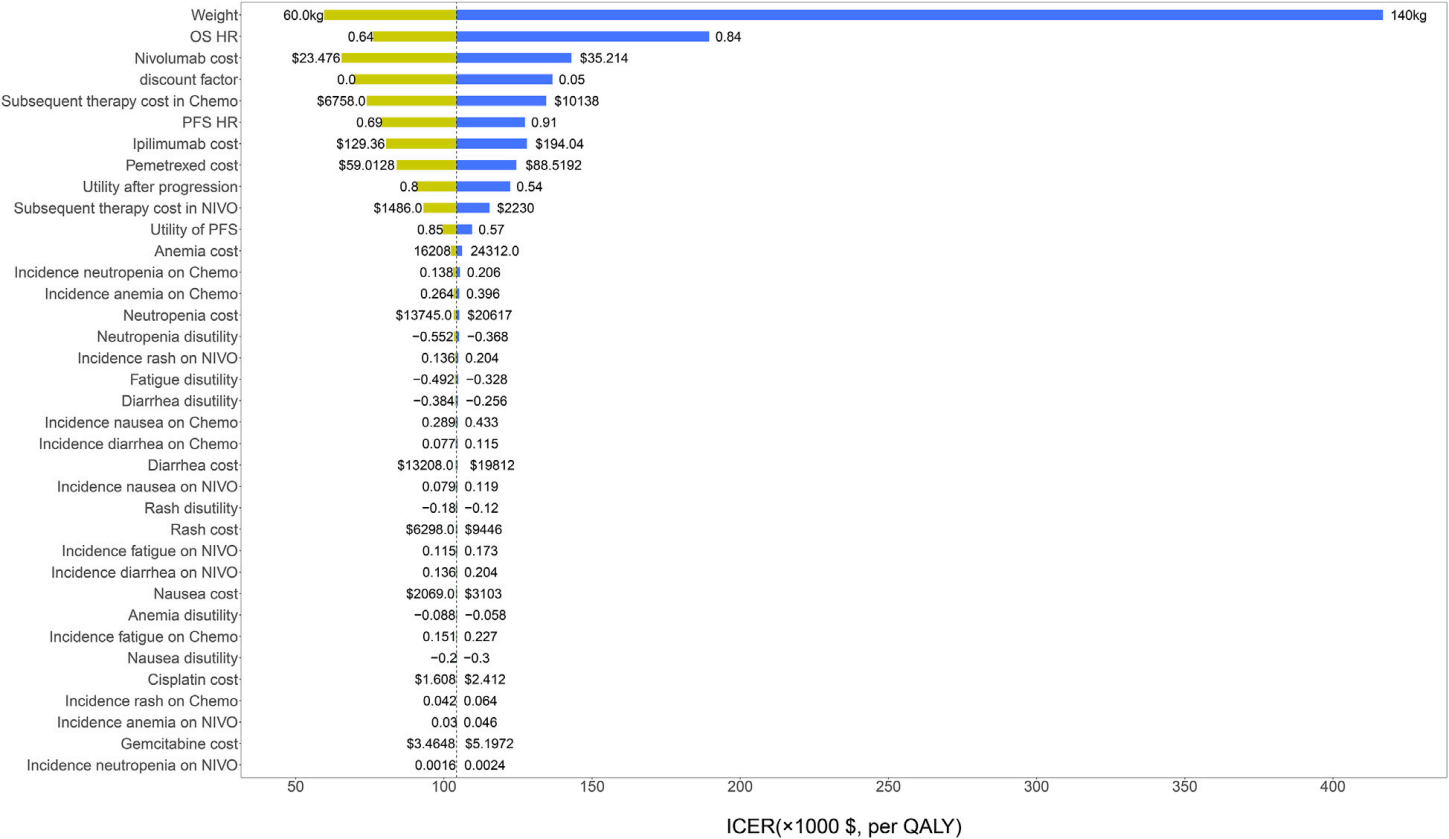
The results of univariable sensitivity analysis. HR, hazard ratio; OS, overall survival; PFS, progression-free survival; NIVO, nivolumab plus ipilimumab; Chemo, chemotherapy; ICER, incremental cost-effectiveness ratio; QALY, quality-adjusted life-years.

The results of univariate sensitivity analyses were shown in the tornado diagram. The variables that had the greatest influence on the ICER were body weight and OS HR. When the patient’s weight increased to 140 kg or the OS HR increased to 0.84, ICER was above the willingness-to-pay (WTP) threshold of $ 150,000/QALY. Other parameters such as drug cost, discount rate and utility value, have a moderate or mild effect on ICER (Figure 2).

**FIGURE 3:**
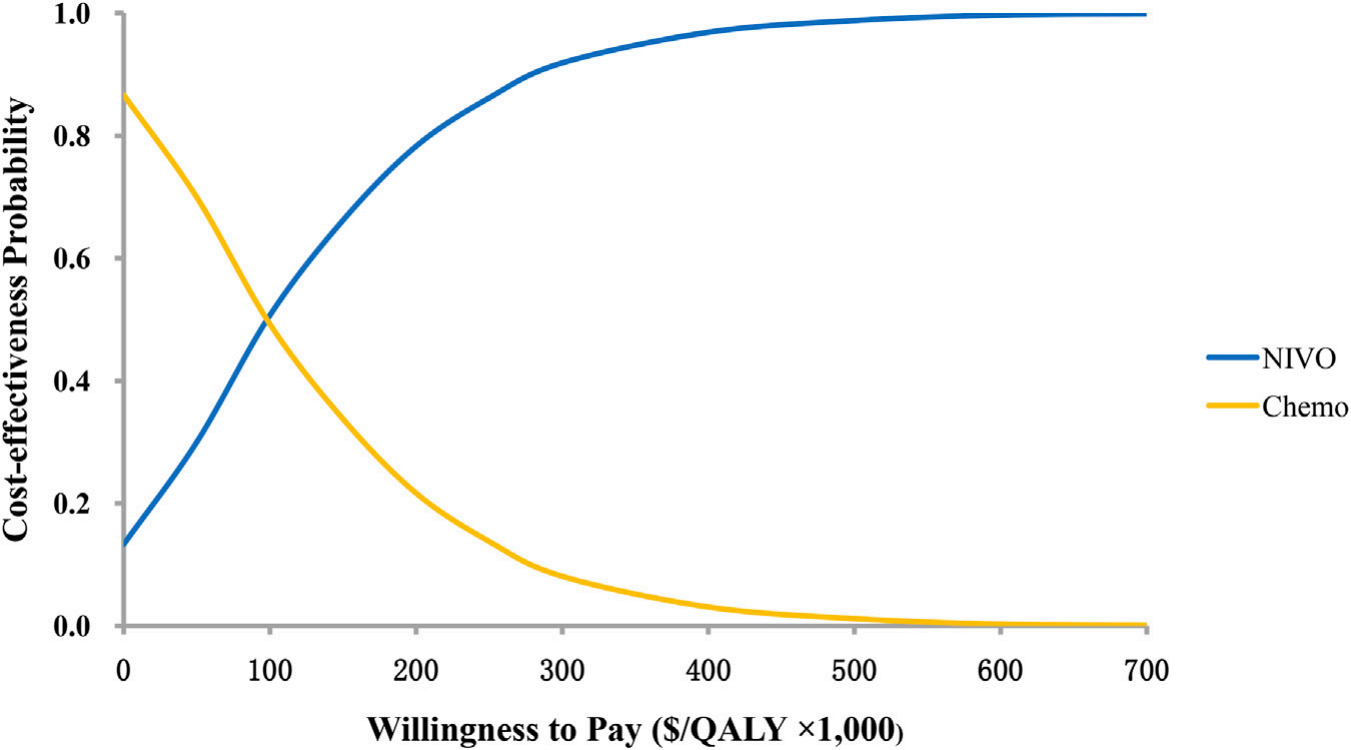
Cost-effectiveness acceptability curves. QALY, quality-adjusted life-years.

The results of the probability sensitivity analysis were shown by the cost-effectiveness acceptability curve (Figure 3). It can be seen from the figure that the probability of nivolumab plus ipilimumab being cost-effectiveness compared to chemotherapy is 50.7 and 66.2% when the WTP value is $ 100,000 and$ 150,000 per QALY.

The results of subgroup analyses showed the ICER remained below $150,000/QALY regardless of the PD-L1 expression level.

## Discussion

To our knowledge, this study is the first cost-effectiveness analysis of nivolumab plus ipilimumab versus chemotherapy as the first-line treatment of advanced NSCLC. Based on our model, nivolumab plus ipilimumab was estimated at $104238 per QALY compared with chemotherapy. The probabilistic sensitivity analyses showed that nivolumab plus ipilimumab was cost-effective at a WTP threshold of $100,000/QALY to $150,000/QALY.

In the past few decades, new anti-cancer drugs have developed rapidly. From the perspective of patients, the high price of anti-cancer drugs may expose cancer patients to serious economic risks, that is, the economic burden caused by medical expenses not covered by medical insurance (Carrera et al., 2018). A new anti-cancer drug costs more than $ 100,000 per year, and medical expenses have become the biggest cause of personal bankruptcy (Mailankody and Prasad, 2015). It is also important for the health care system to cope with extreme medical costs to ensure that patients receive better treatment and minimize economic losses (de Souza and Conti, 2017).

Three previous studies have evaluated the cost-effectiveness of nivolumab plus ipilimumab as a first-line treatment. However, only our current study is to evaluate the cost-effectiveness of nivolumab plus ipilimumab in the treatment of advanced NSCLC. Wu et al. considered the US, UK, and China frameworks and proved that nivolumab plus ipilimumab was cost-effective for the patient with advanced renal-cell carcinoma (RCC) in the UK and the US but not in China (Wu et al., 2018). Our previous study evaluated the cost-effectiveness of nivolumab plus ipilimumab in the US and found that nivolumab plus ipilimumab was a cost-effective treatment for intermediate-and poor-risk patients with metastatic RCC, based on a threshold of $ 100,000 to 150,000 per QALY (Wan et al., 2019b). There is also a Canadian-based study that evaluated the cost-effectiveness of nivolumab plus ipilimumab in the treatment of advanced melanoma, and the results show that this regimen is cost-effective compared with other immunotherapies (Quon et al., 2019).

One factor influencing our model the most was body weight. One our previous study and a study by Wu et al. (2018); Wan et al., 2019b also showed that average body weight had the greatest impact on the ICER of nivolumab plus ipilimumab in patients with advanced RCC in the US. The underlying reason may be that the dose of chemotherapy is not related to body weight, while the dose of nivolumab and ipilimumab needs to be calculated based on body weight. Heavier patients require more doses of nivolumab plus ipilimumab, which may exceed the patient’s affordability.

Our research also has some limitations. First, our cost-effectiveness study is based on specific clinical trials, which are not as extensive and dynamic as the real-world clinical scenario. Second, the use of Log-logistic function to model and predict long-term PFS and OS beyond the experimental observation time is also one of the limitations of this study. Third, we use Medicare reimbursement to estimate the cost of nivolumab plus ipilimumab in the model. In the United States, alternative commercial reimbursement may be higher than Medicare reimbursement. However, due to the lack of public sources of commercial drug cost data, commercial reimbursement cannot be applied to cost-effectiveness analysis.

In conclusion, nivolumab plus ipilimumab as a first-line treatment of advanced NSCLC compared with chemotherapy was estimated to be cost-effective at a WTP threshold of 100,000/QALY to 150,000/QALY from the perspective of US payers.

## Data Availability

All datasets presented in this study are included in the article/Supplementary Material.
