# Supplementary material for: Cost-Effectiveness Analysis of Nivolumab Plus Ipilimumab for Advanced Non-Small-Cell Lung Cancer
Source: Front Pharmacol. 2021 Aug 23;12:580459. doi: 10.3389/fphar.2021.580459 (PMC8430394; doi:10.3389/fphar.2021.580459)
Supplement: Supplementary file 1 [file Table1.DOCX]

**Supplemental Table 1** Background mortality rate

| **Age** | **Background mortality rate** | **Age** | **Background mortality rate** | **Age** | **Background mortality rate** |
| --- | --- | --- | --- | --- | --- |
| 26 | 0.000968 | 51 | 0.004484 | 76 | 0.03287 |
| 27 | 0.000994 | 52 | 0.004874 | 77 | 0.036315 |
| 28 | 0.001024 | 53 | 0.005302 | 78 | 0.040253 |
| 29 | 0.001058 | 54 | 0.005771 | 79 | 0.044908 |
| 30 | 0.001095 | 55 | 0.006274 | 80 | 0.049974 |
| 31 | 0.001132 | 56 | 0.006793 | 81 | 0.055475 |
| 32 | 0.001171 | 57 | 0.007321 | 82 | 0.061509 |
| 33 | 0.001213 | 58 | 0.007854 | 83 | 0.068675 |
| 34 | 0.00126 | 59 | 0.008403 | 84 | 0.076701 |
| 35 | 0.001319 | 60 | 0.008999 | 85 | 0.085469 |
| 36 | 0.001389 | 61 | 0.009652 | 86 | 0.095935 |
| 37 | 0.001467 | 62 | 0.010341 | 87 | 0.107533 |
| 38 | 0.00155 | 63 | 0.011056 | 88 | 0.120347 |
| 39 | 0.001639 | 64 | 0.011804 | 89 | 0.134457 |
| 40 | 0.001743 | 65 | 0.012598 | 90 | 0.149939 |
| 41 | 0.001864 | 66 | 0.013484 | 91 | 0.166861 |
| 42 | 0.002001 | 67 | 0.014501 | 92 | 0.185276 |
| 43 | 0.002159 | 68 | 0.015701 | 93 | 0.205223 |
| 44 | 0.002345 | 69 | 0.017146 | 94 | 0.226719 |
| 45 | 0.002547 | 70 | 0.018855 | 95 | 0.24976 |
| 46 | 0.002778 | 71 | 0.020762 | 96 | 0.274312 |
| 47 | 0.003059 | 72 | 0.022816 | 97 | 0.300311 |
| 48 | 0.003391 | 73 | 0.02501 | 98 | 0.327661 |
| 49 | 0.003753 | 74 | 0.027353 | 99 | 0.356235 |
| 50 | 0.004118 | 75 | 0.029897 | 100 | 1 |
